# Outbreak of COVID-19 in a family, Wenzhou, China

**DOI:** 10.1017/S0950268820001089

**Published:** 2020-05-20

**Authors:** Zu-Mu Zhou, Hai-Zhen Zhou, Xian-Dan Lin, Zhi-Cheng SU, Lu-Shuang Zhao, Xi Chen

**Affiliations:** 1 Wenzhou Center for Disease Control and Prevention, Wenzhou, Zhejiang 325000, China; 2 Wencheng County Center for Disease Control and Prevention, Zhejiang 325300, China

**Keywords:** Coronavirus, COVID-19, investigation, outbreaks

## Abstract

Since December 2019, China has experienced a widespread outbreak of COVID-19. However, at the early stage of outbreak, investigations revealed a variety of patterns resulting in the transmission of COVID-19. Thus, it is essential to understand the transmission types and the potential for sustained human-to-human transmission. Moreover, the information regarding the characteristics of transmission helps in coordinating the current screening programme, and controlling and containing measures, and also, helps in deciding the appropriate quarantine duration. Thus, this investigation reports an outbreak of COVID-19 in a family residing in Wenzhou, Zhejiang, China during the month of January−February 2020.

## Introduction

On 31 December 2019, a novel coronavirus was first suspected and reported in Wuhan, China. Subsequently, on 7 January 2020, this coronavirus, termed as SARS-CoV-2, was identified as the cause of an outbreak of viral pneumonia (COVID-19) in Wuhan [1, 2]. The outbreak has resulted in a global epidemic with a number of confirmed cases of COVID-19 being reported in China and abroad, and new cases are being diagnosed daily [3–7]. On 31 January 2020, the World Health Organization (WHO) declared the outbreak of COVID-19 as a public health emergency of international concern (PHEIC) [8]. In China, during the initial stage of the outbreak, human-to-human transmission of COVID-19 was observed and it included family clusters and healthcare settings [9–13]. This human-to-human transmission led to accelerated spread of COVID-19, ultimately resulting in intercity spread [12]. During the spring festival, a patient with COVID-19, from city X, visited her family living in village A, located in the southeast of Zhejiang province, China. This resulted in a familial outbreak of COVID-19. This paper reports the epidemiological features of an outbreak of COVID-19 in a family of one asymptomatic and eight symptomatic individuals residing in village A, Wenzhou, China.

## Methods

### Case definition

Definitions of suspected case and confirmed case: patients with any two of the following clinical features and any epidemiological risk were the suspected case: (1) clinical features: fever, imaging features of pneumonia, normal or reduced white blood cell count, or reduced lymphocyte count in the early stages of the disease onset. (2) Epidemiologic risk: a history of travel to or residence in Wuhan city, China or other cities with continuous transmission of local cases in the last 14 days before symptom onset; contact with patients with fever or respiratory symptoms from Wuhan city, China or other cities with continuous transmission of local cases in the last 14 days before symptom onset; or epidemiologically connected to SARS-CoV-2 infections or clustered onsets. Suspected case with positive evidence for the SARS-CoV-2 by the real-time PCR test for nucleic acid in respiratory or blood samples is the confirmed case [14].

Definitions of close contacts: those who have one of the following contacts after the onset of confirmed cases in the absence of effective protection [14]: (1) those who live, study, work or have close contact with the confirmed cases, or other close contacts such as working closely with or sharing the same classroom or living in the same house with the confirmed case. (2) Medical and nursing staff and their family members living with them, who treated, nursed or visited the confirmed case, or other personnel who have similar close contact with the case, such as providing direct treatment or care of the case, visiting the case or staying in a closed environment where the cases are located; other patients or caregivers in the same room with case. (3) People who have close contact with the patients in the same transport vehicle, including those who had taken care of the patients on the vehicle; the person who had accompanied the patients (family members, colleagues, friends etc.); other passengers and traffic staff considered having likely close contact with the patients by investigation and evaluation.

### Laboratory testing

Throat swabs for patients and contacts were collected. SARS-CoV-2 nucleic acid detection was carried out by the real-time PCR method.

As this COVID-19 family cluster was an emergency public health issue, an ethics review was not carried out.

## Results

### Index case finding

On 29 January 2020, a 46-year-old female, the index case, developed fever with chills, dry cough, stuffy and runny nose and headache. On the next day, she visited a doctor in clinic and was prescribed anti-cold medication. However, the symptoms did not improve. On the evening of 31 January, she was sent to the emergency department of the county hospital for further treatment. On admission, her body temperature was 38.7 °C. Routine blood examination revealed a white blood cell count of 10.16 × 10^9^/l, neutrocyte ratio of 78.2%, neutrocyte count of 7.94 × 10^9^/l, lymphocyte ratio of 10.3%, and lymphocyte count of 1.05 × 10^9^/l. Her C-reactive protein (CRP) was found to be 33.66/l.

Chest radiography on CT revealed an infectious focus involving the lower lobes of both the lungs, especially the right lung. There was a transmittance of light from the lungs, and increased intensity of patchy nodular density shadow with fuzzy borders of lower lobes of both the lungs. The abnormal density shadows were observed in the other areas of the lungs. The lymph nodes in mediastinum were found to be enlarged. The cardiac silhouette was not found to be obviously abnormal. Moreover, there was no presence of pleural effusions.

She was engaged in a wholesale indoor business in city X, China. She had no history of comorbidities such as hypertension, diabetes or chronic hepatopathy. Neither she had a history of travel to Wuhan, nor attended a dinner party. However, on 22 January 2020, she had a train journey travelling for >24 h from city X to Wenzhou, with her husband. On enquiring, she recalled that during the journey, an unrecognised man occupying the lower berth of the same coach was coughing persistently. The man looked weak and was coughing without putting a mask. She stayed with this unrecognised man for 16 h during travel, while an epidemic of COVID-19 was spreading in many provinces of China. On 24 January, she arrived at Wenzhou and then travelled further to village A by an earliest available bus.

On 31 January 2020, at around 14:00 h, the county hospital reported a suspected patient, the index case, with COVID-19 to the local Center for Disease Control and Prevention and collected throat swabs that were sent to local CDC for SARS-CoV-2 nucleic acid testing, i.e. polymerase chain reaction (PCR). On the next day, at 18:50 h, the throat swabs were found to be positive.

As the patient had high fever, and her symptoms did not improve, on 3 February, at around 13:00 h, she was referred to the First Affiliated Hospital of Wenzhou Medical University for further treatment.

### Outbreak detection and investigation

After the index case was reported, an epidemiological investigation involving the index case, her family members and their contacts was carried out.

After going home to village A on 24 January and before the onset of symptoms of COVID-19, the index patient lived with her family. The family consisted of 14 members, including her husband, parents, elder sister, sister-in-law, brother-in-law, aunt, uncle, younger brother, son, niece and two nephews, which is the same size and structure as most families in the area. Further, she denied having gone outside, and was living and dining with family members. During Spring Festival or Lunar New Year, they had a joyous gathering in village A.

**Fig. 1.**
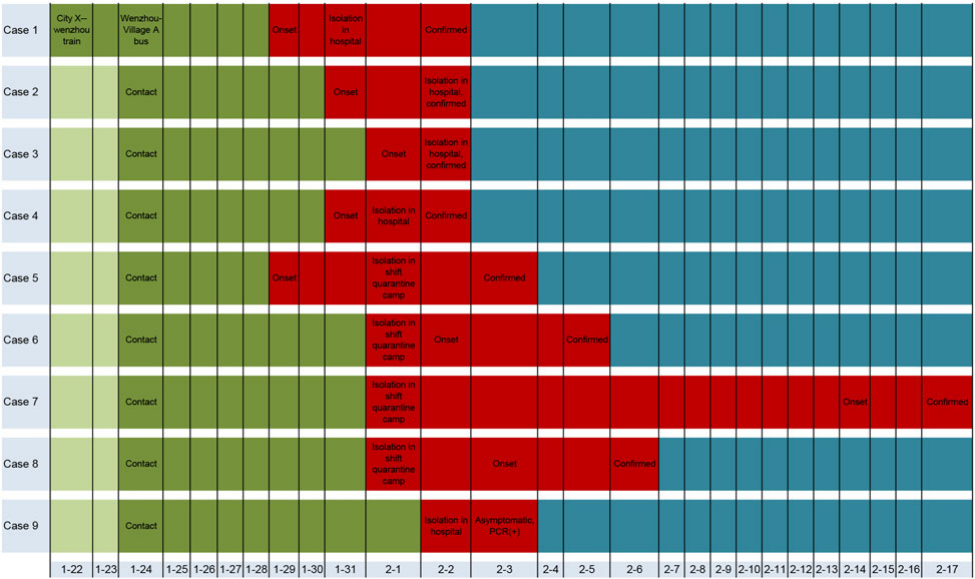
The time series of a family outbreak of COVID-19 in Wenzhou, China.

**Fig. 2.**
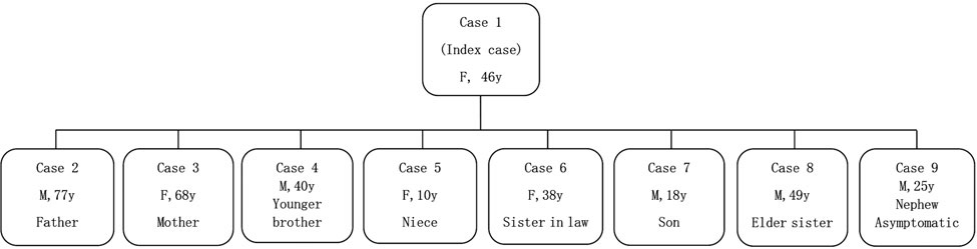
Relations of confirmed cases with COVID-19 pneumonia in a family outbreak.

Search for source of infection, screening of the contacts, and medical observation of the contacts were carried out by medical workers, in order to find as many patients as possible. The throat swabs of contacts were collected and PCR for SARS-CoV-2 nucleic acid was carried out, for all family members, by skilled laboratory workers in the local CDC. All cases were laboratory confirmed, following the case definition by National Health Commission of China. Thirteen family members, 15 close contacts and 10 bus contacts (six contacts in Wenzhou and four in village A) of the index case were traced and found. The onset of symptoms in contacts was one after the other. The time series of a family outbreak of COVID-19 is shown in Figure 1. The investigation confirmed one asymptomatic and seven symptomatic cases with COVID-19 and all of them were family members of the index case. The relations of cases with COVID-19 in a family outbreak are shown in Figure 2. The date of onset and clinical presentation of cases with COVID-19, and the relation of family members with the index case is given in Table 1.

**Table 1.** Date of onset and clinical presentation of cases with COVID-19

Case ID	Relation to index case	Gender	Years	Date of onset	Clinical presentations
Index case		Female	46	29 January	Fever (38.7 °C)，chills, dry cough, running nose, stuffy nose, headache, infectious focus in lower lobes of both lungs on CT, C-reactive protein 33.66/l, PCR( + )
Case 2	Father	Male	77	31 January	Fever (37.8 °C), cough, PCR( + )
Case 3	Mother	Female	68	1 February	Cough, infection in the middle lobe of the right lung on CT, PCR( + )
Case 4	Younger brother	Male	40	31 January	Dry cough, infection in the upper lobe of left lung on CT, PCR( + )
Case 5	Niece	Female	10	29 January	Fever (39.8 °C), infection in the middle lobe of the right lung on CT, PCR( + )
Case 6	Sister in law	Female	38	2 February	Fever (37.7 °C), infection in the middle lobe of the right lung on CT, PCR( + )
Case 7	Son	Male	18	14 February	Fever (38.2 °C), Cough, muscle and joint pain, infection in the upper lobe of the left lung on CT, PCR( + )
Case 8	Elder sister	Female	49	3 February	Fever (37.3 °C), sore throat, cough, infection of the lower lobe of right lung on CT, PCR( + )
Case 9	Nephew	Male	25		Asymptomatic, PCR( + )

However, after 14 days of medical observation, fever and the other related symptoms of COVID-19 were not observed in 15 close contact and 10 bus contacts isolated in individual rooms.

### Outbreak control measures

1. All the patients were admitted in the Department of Infectious Disease of the county hospital for treatment and isolation. The index case and other eight cases were reported to local CDC and public health authority via the web, immediately after being diagnosed as a case of COVID-19.
2. All the close contacts of nine cases were isolated for 14 days, and observed for fever and respiratory symptoms by the healthcare workers in a designated hotel. These workers were instructed to report to a doctor immediately, as soon as the symptoms of discomfort occurred, and take the throat swabs for PCR for SARS-CoV-2 nucleic acid.
3. A collaborative inspection system was initiated to screen the exposed travellers by train from city X on 22–24 January and by buses from Wenzhou and village A on 24 January. All the exposed travellers (six and four exposed individuals in the same bus in Wenzhou and village A, respectively; and no close contacts were found amongst traced individuals in train) were advised self-isolation and they stayed at home for 14 days.
4. Thorough screening and tracing activity resulted in finding 185 contacts of the index cases, in village A. Out of these 185 contacts, 58 and 127 contacts were isolated in a shift quarantine camps and at home, respectively. On 4 February, 1230 individuals living in 366 houses located in the residential district of the index case were subjected to medical observation at home. These contacts were followed up for the entire period of 14 days. Despite active follow-up, no secondary transmission event were detected.
5. The agriculture trade market neighbouring the patient's house was closed, so as to decrease the flow and the contact of people.
6. Individuals coming in contact with COVID-19 cases and their close contacts were asked to frequently wash the hands, open the windows so as to increase the ventilation and put on masks.
7. The terminal disinfection of the focus, the building, and sites of living and activities of the patients were carried out with chlorine-containing disinfectants.

## Discussion

The ongoing COVID-19 outbreak has rapidly evolved and spread globally [15]. Epidemiological studies suggest that SARS-CoV-2 has an intrinsic capacity to cause epidemic spread [16]. SARS-CoV-2 appears to be highly transmissible from human-to-human resulting in a wide spectrum of clinical manifestations in patients with COVID-19 [15]. However, similar to our investigation, increasing evidence suggests that mild clinical symptoms could be more frequent in cases of COVID-19. In our investigation, amongst nine cases, one had severe symptoms, seven had mild symptoms and one was asymptomatic.

Although the Huanan market in Wuhan was highly relevant to the emergence and spread of COVID-19, and the SARS-CoV-2 has been detected in environmental samples obtained from the market, the origin of this virus has been determined conclusively [17]. Currently, there is no confirmed animal reservoir of the virus, but the available evidence strongly supports that the SARS-CoV-2 was derived from bats [7, 18]. However, it is not yet clear whether SARS-CoV-2 infected humans via direct transmission from a bat or through an intermediate host [17]. It is believed that clarifying the source of the virus will help determine zoonotic transmission patterns. Notably, cases have occurred in patients who had not travelled to Wuhan, thereby, indicative of human-to-human transmission [19]. Moreover, various investigations have resulted in a positive evidence supporting human-to-human transmission of SARS-CoV-2.

Similar human-to-human transmission was observed in our investigation. The index patient had the history of contact with the suspected patient and the obvious clinical symptoms of COVID-19. Moreover, the test results of SARS-CoV-2 nucleic acid of throat swabs were positive. Therefore, the index case was diagnosed as COVID-19, following the case definition by National Health Commission of China [14].

The investigation revealed that the index case had 14 family members. The index case did not contact other people with similar symptoms before the onset of symptoms, except the suspected patient occupying the lower berth of the same coach of the train. The suspected patient had continuous cough and they travelled together for 16 h. In addition, transmission of COVID-19 occurs easily in closed coaches. Thus, it is recognised that the source of infection for the index case is this suspected patient.

After arriving at village A, before the onset of symptoms (i.e. from 24–29 January), the index case had been dwelling with her family members. They had meals and some activities together, in a four-floor building with six bedrooms. All the family members, including the index case had not gone outside, before the onset of symptoms in the index case. During this period, they did not have any visitors. Our investigation revealed that they did not come in contact with people from Wuhan or individuals with similar symptoms. However, all seven symptomatic and one asymptomatic cases had developed a contact with the index case. Thus, we believed that the source of infection for these eight cases was the index case. Moreover, this direct human-to-human transmission of COVID-19 amongst the family members was due to close contact with the index case and they developed the symptoms one after another. Fifteen close contacts except the family members did not develop COVID-19. Thus, over-crowding as a risk factor can also play a significant role in the transmission of COVID-19. The high infectivity of COVID-19 was discovered from the investigation of this family outbreak leading to development of symptoms in eight patients and one infected asymptomatic individual.

The range of incubation period is required for the purpose of epidemiological case definitions, and is essential to determine the appropriate duration of quarantine [20]. Moreover, the knowledge of the incubation period helps in assessing the effectiveness of entry screening and contact tracing programme. The distribution of incubation period is also used in estimating the size of the epidemic [21, 22] and the transmission potential [23]. During the early outbreak phase, the mean incubation period, in Wuhan, was 5.2 days (95% confidence interval (CI), 4.1–7.0), with the 95th percentile of the distribution at 12.5 days [13]. In our investigation, however, the median incubation period of COVID-19 was 7.5 days, with the longest and shortest incubation periods being 21 and 5 days, respectively. The patient with the longest incubation period was the 18-year-old son of the index case. On or after 24 January, he developed a contact with the index case, who was asymptomatic before 29 January. After 21 days, he presented with fever (38.2 °C), cough, myalgia and arthralgia. Chest radiography on CT revealed an infectious focus involving the upper lobe of the left lung. The throat swabs of the patient were collected and examined, and were found to be positive for SARS-CoV-2 nucleic acid. Moreover, he had been isolated when the index case was diagnosed with COVID-19. It is important to note that such a long incubation period of COVID-19 is very rare in literature.

Our investigation indicates that the cases are highly infectious even during the incubation period. Moreover, it has been demonstrated that the familial outbreak of COVID-19 has a potential for human-to-human transmission, even during the incubation period. This is supported by our investigation, where the index case during the incubation period transmitted COVID-19 to one asymptomatic and seven symptomatic cases. Thus, it is prudent to consider this fact while carrying out an epidemiological investigation and taking control measures against COVID-19, in future, including a quarantine and observation period for the suspected cases. Moreover, it is judicious to consider an incubation period, as long as 21 days, as highlighted by our investigation.
